# COX-2 Gene Promoter Polymorphism and Coronary Artery Disease in Middle-Aged Men: The Helsinki Sudden Death Study

**DOI:** 10.1155/2008/289453

**Published:** 2008-03-11

**Authors:** Kati H. Huuskonen, Tarja A. Kunnas, Minna M. Tanner, Jussi Mikkelsson, Erkki Ilveskoski, Pekka J. Karhunen, Seppo T. Nikkari

**Affiliations:** ^1^Department of Medical Biochemistry, Medical School, University of Tampere, 33104 Tampere, Finland; ^2^Laboratory of Cancer Genetics, Institute of Medical Technology, University of Tampere, 33104 Tampere, Finland; ^3^Department of Forensic Medicine, Medical School, University of Tampere, 33104 Tampere, Finland; ^4^Heart Center, Tampere University Hospital, P.O. Box 2000, 33521 Tampere, Finland; ^5^Research Unit of the Laboratory Centre, Tampere University Hospital, P.O. Box 2000, 33521 Tampere, Finland

## Abstract

Cyclooxygenase (COX) catalyzes formation of prostaglandins that contribute to the inflammation in atherosclerosis. Our objective was to study whether the functional C variant of the −765G→C polymorphism in the human COX-2 gene associates with the severity of coronary atherosclerosis measured at the coronary artery level. The Helsinki sudden death study autopsy material (*n* = 300) comprised of Finnish men who died suddenly. The area of atherosclerotic lesions in the coronary arteries was quantitated, and coronary narrowing was measured. The occurrence of myocardial infarction (MI) was assessed. Genotyping was by restriction endonuclease analysis. Men carrying the minor C allele had larger areas of complicated lesions (*P* = .024) and a higher number of coronary arteries that had over 50% stenosis (*P* = .036) compared to men representing the common GG genotype. The COX-2 polymorphism was not associated with MI. Our data suggest that COX-2 may be involved in plaque growth.

## 1. INTRODUCTION

Cyclooxygenase (COX) catalyzes the first two steps in prostanoid production from arachidonic acid to many prostaglandins including prostacyclin and thromboxane. The enzyme has three known isoforms. COX-1 is constitutively expressed in most human tissues under basal conditions. COX-2 expression is primarily induced in response to inflammatory stimuli by growth factors, mitogens, and cytokines [1]. COX-3 is a COX-1 derived isozyme. Its functional role still remains poorly understood [2].

COX-2 levels are raised in chronic inflammatory diseases including atherosclerosis. COX-2 expression has been detected in endothelial cells, smooth muscle cells, monocytes and macrophages within human atherosclerotic lesions [3, 4]. Many prostaglandins produced by the COX-2 route including thromboxane stimulate vasoconstriction, platelet aggregation, and leukocyte-endothelial cell adhesion, which all contribute to formation of atherosclerosis and thrombosis [5–7]. However, the main prostaglandin produced by endothelial cells is prostacyclin (PGI_2_), which acts as a vasodilator and inhibits platelet aggregation, leukocyte activation, and adhesion [8].

A functional G→C polymorphism located 765 basepairs upstream from the transcription start site (−765G→C) has been identified in the human COX-2 gene with C allele leading to decreased promoter activity in vitro [9]. Previous studies have shown that C allele might provide protective effects against clinical events, for example, myocardial infarction (MI), stroke [10] and cerebrovascular ischemia [11]. C allele may also be associated with lower levels of inflammatory markers such as C-reactive protein and interleukin-6 in cardio/cerebrovascular and hypercholesterolemic patients [9–12]. However, in contrast to these prior data, Hegener et al. found no evidence for an association of the COX-2 polymorphisms/haplotypes neither with risk of incident MI nor with ischemic stroke [13]. Furthermore, Kohsaka et al. have recently reported that the COX-2-765G→C polymorphism is in fact a risk factor for incident stroke in African-Americans [14].

The previous contradictory observations suggest that additional evaluation is warranted to investigate the association between the COX-2 765G→C polymorphism and cardiovascular disease. We have examined the association of this polymorphism with the risk of severity of atherosclerosis at the coronary artery level in a previously collected autopsy series of a genetically homogeneous population of Finnish men who had died suddenly out of hospital.

## 2. SUBJECTS AND METHODS

### 2.1. Subjects

HSDS was designed to investigate factors predisposing to sudden death in Finnish middle-aged men living in Helsinki and its environment [15]. The autopsy series was collected during 12 months in 1991-1992 at the department of Forensic Medicine in the University of Helsinki. The indications for autopsy were out-of-hospital death of a previously healthy person, accidental death, suspected intoxication, suicide, and death in connection with medical treatment. The original study population comprised a prospective series of 300 males aged 33–70 years (mean 53 years). This autopsy series covered 28% of all deaths of males within this age group in the area of Helsinki during the study period in question. The cause of death was cardiac disease (coronary heart disease (CHD), cardiomyopathies, hypertrophy, or dilatation of the heart) in 38.3% (*n* = 115), other diseases in 20.3% (*n* = 61) and violent death (suicides and accidents) in 41.4% (*n* = 124). Men with a coronary event (MI, AMI with coronary thrombus, or coronary thrombus only) (*n* = 72) were also compared with men with no coronary event (*n* = 228).

### 2.2. Measurements of coronary artery disease

At autopsy, coronary angiography was performed using vulcanizing liquid silicone rubber mixed with lead oxide as contrast medium [16]. Proximal, middle, and distal narrowing of the main trunks of the left anterior descending artery (LAD), right coronary artery (RCA), and left circumflex artery (LCX) were measured with a micrometer on the rubber cast model. The percentage of coronary narrowing was obtained by dividing the diameter (in millimeters) of the greatest stenosis by that of the nearest proximal unaffected part of the cast model of the same artery. Based on the presence of over 50% stenosis in one, two, or three major coronary arteries, the study population was divided into subgroups according to number of diseased vessels.

The proximal parts of the LAD, LCX, and RCA were dissected free, opened, and attached to a card and then fixed in 10% buffered formalin. The vessel wall was subsequently stained for fat by the Sudan IV method. The following atherosclerotic changes were assessed: fatty streak, fibrous plaque, complicated lesion, and the area of calcification. Flat or raised intimal lesions that were distinctly stained by Sudan IV and did not show more complex changes were classified as fatty streaks. An elevated lesion that did not display ulceration, haemorrhage, necrosis, or thrombosis was considered as a fibrous plaque. An area was regarded as complicated lesion, if it expressed one or several changes mentioned above, with or without calcium deposit. The part of the aorta showing intense X-ray-positive signal in the radiogram was considered as an area of calcification. The areas of atherosclerotic lesions and the total areas of coronary segments were evaluated using the standard planimetric technique [15]. The proportions of the divergent atheromatous changes were calculated based on the total surface area of the coronary arteries [15]. The occurrence of MI in the series was confirmed by a macroscopic and histologic examination of the myocardium. The presence of coronary thrombus was recorded during autopsy when coronary arteries were dissected longitudinally.

Autopsy and COX-2 genotype data were available in 300 cases, these comprising the final study population.

### 2.3. Risk factors underlying coronary artery disease

A spouse, relative, or a close friend of the deceased was interviewed within 2 weeks following the autopsy. Besides the questions pertaining to risk of sudden death (i.e., arterial hypertension, diabetes), additional questions were included to define the smoking habits of the deceased. The relative/friend was asked whether the person had smoked during his life and how many cigarettes he had smoked daily. Data on smoking habits were obtained in 148 cases. In addition to COX-2 genotype and autopsy data, complete data on all risk factors were available in 118 cases.

### 2.4. COX-2 genotyping

The promoter region of the human COX-2 gene surrounding the site of −765G→C polymorphism was polymerase chain reaction (PCR) amplified using DNAs extracted from cardiac muscle as a template. Primers for DNA amplification were designed based on the published sequence of the human COX-2 gene (NCBI/U04636, gi: 496975) using the Primer3 software (http://frodo.wi.mit.edu/cgi-bin/primer3/primer3_www.cgi). The 25 *μ*l-reaction was composed of 50 pmol of each primer (TAG Copenhagen, Copenhagen, Denmark) (forward 5′ -CATTAACTATTTACAGGGTAACTGCTT-3′; reverse 5′-TGCAGCACATACATACATAGCTTTT-3′), 200 *μ*M of each dNTP and 2,5 U HotStarTaq DNA Polymerase in 1 × PCR buffer (Qiagen, Valencia, CA, USA). PCR conditions included 15 minutes of initial polymerase activation step at 94^°^C followed by 35 three-step cycles of denaturation at 94^°^C for 30 seconds, annealing at 56^°^C for 30 seconds, extension at 72^°^C for 30 seconds, and final extension at 72^°^C for 5 minutes.

The primers generated fragments of 228 bp which were genotyped by SsiI (Fermentas, Vilnius, Lithuania) restriction endonuclease. PCR product was cleaved into fragments of 168 bp and 60 bp, if the G allele was present. Digested products were resolved with 2% MetaPhor (Cambrex, East Rutherford, NJ, USA) agarose gel electrophoresis and visualized by ethidium bromide staining.

### 2.5. Statistical analysis

Results were analyzed by SPSS for Windows software, version 14.0 (SPSS, Chicago, IL, USA). Mean ± standard error (SE) is reported for continuous variables. For normally distributed continuous variables, one-way-ANCOVA was used with age, body mass index (BMI), smoking status (yes/no), hypertension (yes/no), and diabetes (yes/no) as covariates. Since variables measuring atherosclerotic changes were not normally distributed, logarithmic transformations were utilized for values of fatty streaks, fibrous plaques, calcifications, and complicated lesions, but results are displayed in crude form.

## 3. RESULTS

In the study population, COX-2 genotype frequencies were GG 74.4%, GC 24.3%, and CC 1.3%. Since there were only four cases representing the CC genotype, men carrying the C allele were pooled together as one group for statistical analyses.

**Figure 1:**
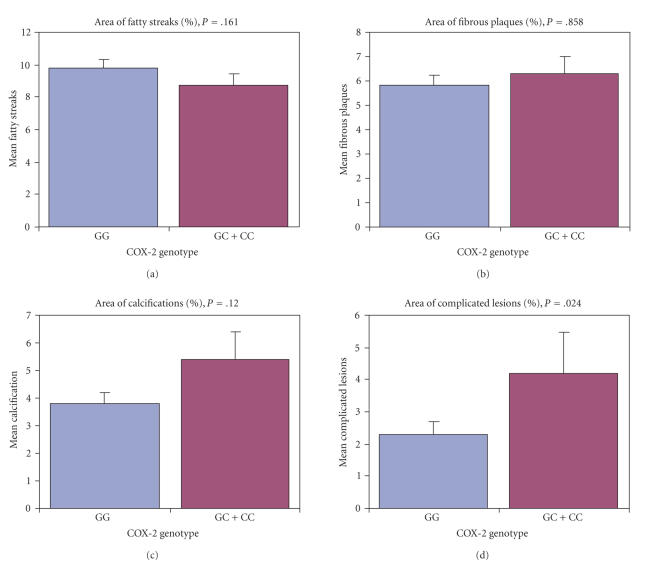
Association of COX-2 genotype with atherosclerotic changes. *P* values are from ANCOVA with age and BMI as covariates. Error bars represent SE.

Men carrying the C allele had significantly larger areas of complicated lesions in their coronary arteries than the men with GG genotype (*P* = .024) with adjustment for age and BMI. After further adjustment with smoking, hypertension, and diabetes, there was a significant difference between genotypes and calcifications (*P* = .031) and genotypes and complicated lesions (*P* = .017) (Table 1, Figure 1). No significant differences existed between COX-2 genotypes and the areas of fibrous plaques or fatty streaks. The C allele carriers had a higher number of over 50% stenosis in one, two, or three major coronary arteries (*P* = .036) compared to those with the GG genotype, with adjustment for age and BMI. After further adjustment with smoking, hypertension, and diabetes, this difference was even more significant (*P* = .008) (Table 1, Figure 1). There was no statistically significant association between genotype and occurrence of MI (acute, old, or any MI) by age, BMI, smoking, hypertension, and diabetes as covariates (Table 1).

Men with a coronary event (MI, AMI with coronary thrombus, or coronary thrombus only) (*n* = 72) were also compared with men with no coronary event (*n* = 228). In the coronary event group, men carrying the C allele had significantly larger areas of complicated lesions in their coronary arteries than the men with GG genotype with adjustment for age and BMI (*P* = .040) and after further adjustment with smoking, hypertension, and diabetes (*P* = .015). Also in the coronary event group, the C allele carriers had a higher number of over 50% stenosis in one, two, or three major coronary arteries compared to those with the GG genotype, with adjustment for age and BMI (*P* = .040) and after further adjustment with smoking, hypertension, and diabetes (*P* = .050) (Table 1). No significant differences existed between COX-2 genotypes and the areas of fibrous plaques or fatty streaks in the coronary event group (data not shown). The genotypes did not differ in the group with no coronary event (Table 1).

## 4. DISCUSSION

The results of this study propose that middle-aged Finnish men with sudden death that carried the rare C allele of the COX-2 765G→C polymorphism had more often advanced coronary plaques, characterized by more extensive areas of complicated lesions, compared to men with the GG genotype. This association was most pronounced in subjects with a coronary event. Variation in COX-2 promoter genotype showed no association with early coronary atherosclerosis characterized by fatty streaks and fibrous plaques. A previous study has shown that the 765G→C allele might provide protective effects against myocardial infarction [10]. C allele may also be associated with lower levels of inflammatory markers such as C-reactive protein and interleukin-6 in cardio/cerebrovascular and hypercholesterolemic patients [9, 12]. In contrast to these prior data, Hegener et al. found no evidence for an association of the polymorphism with risk of incident MI [13]. Furthermore, Kohsaka et al. have recently reported that the 765G→C allele is in fact a risk factor for incident stroke in African-Americans [14]. We did not observe an association of this polymorphism on MI, but a larger sample size than our material is needed to resolve the question. Nevertheless, our observations of complicated atherosclerosis at the coronary artery level suggest that the 765G→C allele is in fact a risk factor for coronary disease. A major limitation of our study was that there were only 118 subjects with complete data on risk factors underlying coronary artery disease. However, this further statistical adjustment strengthened rather than weakened our findings on the original 300 men, adjusted for age and BMI. Nevertheless, a study on a larger sample is necessary to confirm the results of the present investigation.

Men carrying the C allele had a higher number of coronary arteries that had over 50% stenosis, compared to men who were GG homozygous. In contrast, the relation between the C allele and severity of atherosclerosis has previously been studied at the coronary level by angiography in an Italian population, but no association was found [10]. The polymorphism under study seems to be relatively rare in the Finnish population with frequencies of GG 74.4%, GC 24.3%, and CC 1.3% whereas in the Italian population frequencies are GG 50.7%, GC 43.3%, and CC 6.4% [10].

The finding that stenosis was more often observed in men possessing C allele than in men representing GG genotype may provide support for the hypothesis that C allele may lead to more extensive plaque growth. In fact, since the C allele leads to decreased promoter activity [9], it could contribute to lower-prostaglandin production. Matrix metalloproteinases (MMPs) and the extracellular matrix (ECM) degrading enzymes are partly up regulated by PGE_2_ which is generated through COX-2 route. As a consequence of diminished PGE_2_ biosynthesis, a decrease in MMP-9 expression could follow [17] and lead to accumulation of extracellular matrix by smooth muscle cells. In line with our results, Cipollone et al. [10] reported that in carotid plaques of C allele carriers there is more interstitial collagen compared to plaques of GG homozygotes which could contribute to plaque growth [18, 19].

In conclusion, the rare COX-2 gene C allele associated with complicated plaques and severe stenosis at the coronary artery level. This suggests that COX-2 may be involved in plaque growth. No protective effect against MI was seen.

## Figures and Tables

**Table 1:** Association of combined COX-2 genotypes with coronary atherosclerotic changes, number of main coronary arteries with stenosis of over 50%, and myocardial infarction.

	Genotype			
	GG	GC + CC	*P* value*	*P* value^†^
All subjects	*n* = 223	*n* = 77	*n* = 300	*n* = 118
Atherosclerotic changes (%)				
Fatty streaks	9.91 ± 0.54	8.69 ± 0.74	0.161	0.341
Fibrous plaques	5.79 ± 0.41	6.31 ± 0.72	0.858	0.567
Calcifications	3.78 ± 0.39	5.37 ± 1.01	0.120	**0.031**
Complicated lesions	2.24 ± 0.37	4.15 ± 1.25	**0.024**	**0.017**
Number of coronary arteries with stenosis of over 50% (0–3)	0.33 ± 0.67	0.58 ± 0.93	**0.036**	**0.008**
Myocardial infarction	23.3%	26.0%	0.809	0.350

Subjects with coronary event	*n* = 52	*n* = 20	*n* = 72	*n* = 35
Complicated lesions	6.81 ± 8.67	14.00 ± 18.84	**0.040**	**0.015**
Number of coronary arteries with stenosis of over 50% (0–3)	1.00 ± 1.00	1.69 ± 1.14	**0.040**	**0.050**

Subjects with no coronary event	*n* = 172	*n* = 56	*n* = 228	*n* = 83
Complicated lesions	0.91 ± 2.85	0.88 ± 1.55	0.876	0.541
Number of coronary arteries with stenosis of over 50% (0–3)	0.18 ± 0.46	0.20 ± 0.40	0.884	0.990

Mean ± SE. ANCOVA * adjusted by age and BMI, or ^†^ age, BMI, smoking, hypertension and diabetes.

## References

[1] DeWitt D, Smith WL (1995). Yes, but do they still get headaches?. *Cell*.

[2] Chandrasekharan NV, Dai H, Roos KLT (2002). COX-3, a cyclooxygenase-1 variant inhibited by acetaminophen and other analgesic/antipyretic drugs: cloning, structure, and expression. *Proceedings of the National Academy of Sciences of the United States of America*.

[3] Baker CSR, Hall RJC, Evans TJ (1999). Cyclooxygenase-2 is widely expressed in atherosclerotic lesions affecting native and transplanted human coronary arteries and colocalizes with inducible nitric oxide synthase and nitrotyrosine particularly in macrophages. *Arteriosclerosis, Thrombosis, and Vascular Biology*.

[4] Schonbeck U, Sukhova GK, Graber P, Coulter S, Libby P (1999). Augmented expression of cyclooxygenase-2 in human atherosclerotic lesions. *American Journal of Pathology*.

[5] Granstrom E, Diczfalusy U, Hamberg M, Hansson G, Malmsten C, Samuelsson B (1982). Thromboxane a2: biosynthesis and effects on platelets. *Advances in Prostaglandin, Thromboxane, and Leukotriene Research*.

[6] Needleman P, Turk J, Jakschik BA, Morrison AR, Lefkowith JB (1986). Arachidonic acid metabolism. *Annual Review of Biochemistry*.

[7] Linton MF, Fazio S (2004). Cyclooxygenase-2 and inflammation in atherosclerosis.. *Current Opinion in Pharmacology*.

[8] Bunting S, Gryglewski R, Moncada S, Vane JR (1976). Arterial walls generate from prostaglandin endoperoxides a substance (prostaglandin X) which relaxes strips of mesenteric and coeliac arteries and inhibits platelet aggregation. *Prostaglandins*.

[9] Papafili A, Hill MR, Brull DJ (2002). Common promoter variant in cyclooxygenase-2 represses gene expression: evidence of role in acute-phase inflammatory response. *Arteriosclerosis, Thrombosis, and Vascular Biology*.

[10] Cipollone F, Toniato E, Martinotti S (2004). A polymorphism in the cyclooxygenase 2 gene as an inherited protective factor against myocardial infarction and stroke. *The Journal of the American Medical Association*.

[11] Colaizzo D, Fofi L, Tiscia G (2006). The COX-2 G/C-765 polymorphism may modulate the occurrence of cerebrovascular ischemia. *Blood Coagulation and Fibrinolysis*.

[12] Orbe J, Beloqui O, Rodriguez JA, Belzunce MS, Roncal C, Páramo JA (2006). Protective effect of the G-765C COX-2 polymorphism on subclinical atherosclerosis and inflammatory markers in asymptomatic subjects with cardiovascular risk factors. *Clinica Chimica Acta*.

[13] Hegener HH, Diehl KA, Kurth T, Gaziano JM, Ridker PM, Zee RYL (2006). Polymorphisms of prostaglandin-endoperoxide synthase 2 gene, and prostaglandin-E receptor 2 gene, C-reactive protein concentrations and risk of atherothrombosis: a nested case-control approach. *Journal of Thrombosis and Haemostasis*.

[14] Kohsaka S, Volcik KA, Folsom AR (2008). Increased risk of incident stroke associated with the cyclooxygenase 2 (COX-2) G-765C polymorphism in African-Americans: the atherosclerosis risk in communities study. *Atherosclerosis*.

[15] Ilveskoski E, Perola M, Lehtimäki T (1999). Age-dependent association of apolipoprotein E genotype with coronary and aortic atherosclerosis in middle-aged men: an autopsy study. *Circulation*.

[16] Weman SM, Salminen US, Penttilä A, Männikkö A, Karhunen PJ (1999). Post-mortem cast angiography in the diagnostics of graft complications in patients with fatal outcome following coronary artery bypass grafting (CABG). *International Journal of Legal Medicine*.

[17] Libby P, Geng YJ, Aikawa M (1996). Macrophages and atherosclerotic plaque stability. *Current Opinion in Lipidology*.

[18] Libby P (1995). Molecular bases of the acute coronary syndromes. *Circulation*.

[19] Shah PK, Falk E, Badimon JJ (1995). Human monocyte-derived macrophages induce collagen breakdown in fibrous caps of atherosclerotic plaques: potential role of matrix-degrading metalloproteinases and implications for plaque rupture. *Circulation*.

